# Exercise suppresses neuroinflammation for alleviating Alzheimer’s disease

**DOI:** 10.1186/s12974-023-02753-6

**Published:** 2023-03-19

**Authors:** Minghui Wang, Hu Zhang, Jiling Liang, Jielun Huang, Ning Chen

**Affiliations:** Tianjiu Research and Development Center for Exercise Nutrition and Foods, Hubei Key Laboratory of Exercise Training and Monitoring, College of Sports Medicine, Wuhan Sports University, Wuhan, 430079 China

**Keywords:** Alzheimer’s disease, Neuroinflammation, Neurofibrillary tangle, Senile plaque, Exercise

## Abstract

Alzheimer’s disease (AD) is a chronic neurodegenerative disease, with the characteristics of neurofibrillary tangle (NFT) and senile plaque (SP) formation. Although great progresses have been made in clinical trials based on relevant hypotheses, these studies are also accompanied by the emergence of toxic and side effects, and it is an urgent task to explore the underlying mechanisms for the benefits to prevent and treat AD. Herein, based on animal experiments and a few clinical trials, neuroinflammation in AD is characterized by long-term activation of pro-inflammatory microglia and the NOD-, LRR- and pyrin domain-containing protein 3 (NLRP3) inflammasomes. Damaged signals from the periphery and within the brain continuously activate microglia, thus resulting in a constant source of inflammatory responses. The long-term chronic inflammatory response also exacerbates endoplasmic reticulum oxidative stress in microglia, which triggers microglia-dependent immune responses, ultimately leading to the occurrence and deterioration of AD. In this review, we systematically summarized and sorted out that exercise ameliorates AD by directly and indirectly regulating immune response of the central nervous system and promoting hippocampal neurogenesis to provide a new direction for exploring the neuroinflammation activity in AD.

## Background

Alzheimer's disease (AD) is one of the most common neurodegenerative diseases. Nowadays, it is estimated to be approximately 50 million AD patients all over the world, and the number will reach up to 76.14 million in 2030 [1]. Although the understanding of AD concept and pathogenesis has advanced since the first case was reported in 1907 [2], there are no effective treatment strategies. According to the amyloid cascade hypothesis, AD is caused by the excessive accumulation of amyloid-β peptide (Aβ) generated from amyloid precursor protein (APP) in the brain, especially Aβ42 and its polymers. The decreased clearance rate of Aβ is also an essential factor for Aβ deposition [3]. In addition, Aβ also can induce the hyperphosphorylation of microtubule-associated protein Tau [4], which promotes the dissociation of tubulin and the aggregation of tubulin into bundles, thereby resulting in pairs of helical filaments and neurofibrillary tangles (NFTs), and eventually causing neuronal dysfunction and even death [5]. Tau protein deposition is also considered to be another critical pathological feature of AD. During exploring the broad neuropathology of the human brain across the lifespan, the experiments in many primates tell us that the deposition of Aβ may be an aging-related by-product. The abnormal increase in hyperphosphorylation of Tau protein is likely to be the initial cause of AD pathogenesis [6, 7]. Consequently, further exploration of the pathogenesis and treatments of AD is highly desired from other perspectives.

Since neuroinflammation hypothesis was proposed in 1992, neuroinflammation caused by disturbed inflammatory neuroimmune system has become the third core pathological feature of AD [8]. Several studies have demonstrated inflammatory responses accompanied by the activation of immune cells in the brains of early clinical AD patients and postmortem pathological tissues as well as animal models [9–12]. Many antibodies that target these tissues are determined in the cerebrospinal fluid (CSF) of AD patients, indicating that AD is likely to be an inflammatory disease accompanied by autoimmune activation [13]. Recently, the Food and Drug Administration (FDA) has approved sodium oligomannate, a seaweed extract, for the treatment of AD patients in China, which can suppress the neuroinflammatory response by inhibiting the accumulation of phenylalanine and isoleucine in the blood [14]. Moreover, the drug has successfully passed the phase III clinical trial [15]. Although epidemiological studies suggest the applications of non-steroid anti-inflammatory drugs (NSAIDs) to prevent AD, most clinical trials have been unsuccessful [16]. The major reasons may be correlated with the precision targets [17], blood-brain barrier [18], adverse reactions [19], and medication timing [20]. It is worth noting that more and more studies have confirmed that scientific and reasonable exercise can effectively regulate the neuroimmune system, which has been proven in cardiovascular disease, respiratory system, and other diseases [21, 22]. Relevant animal and clinical experiments have also demonstrated that exercise can alleviate the symptoms of neurodegenerative diseases and delay the pathological progression in various ways [23], indicating that there is a close connection between exercise and the immune system [24]. In recent years, exercise has gradually received extensive attention in preventing and treating AD. Therefore, we systematically summarized the relationships among exercise, neuroinflammation and AD, which will provide relevant theoretical references for the suppression of neuroinflammation to realize the prevention and treatment of AD upon exercise interventions.

## A new perspective on AD-related hypotheses

AD is an age-dependent neurodegenerative disease accompanied by cognitive impairment, memory loss, and abnormal behavior [3]. From the cholinergic hypothesis proposed in 1976 [25], to the amyloid cascade hypothesis in 1991 [26], and even to the recent hypotheses with cellular aging [27] and dysfunctional immune regulation [28], a large number of scholars have contributed academic explanation of AD pathogenesis. Over the past three decades, many experimental studies have documented that Aβ is the culprit of neurodegeneration in AD [26, 29], thereby confirming the deposition of Aβ and the accumulation of hyperphosphorylated Tau protein in neurons as the major pathogenesis of AD to lead to impaired synaptic plasticity and cognitive dysfunction, as well as the occurrence of dementia. Due to the complexity of pathological process of AD, a single hypothesis could not fully clarify the specific pathogenesis of AD, which also provides the explanation for failed clinical trials of drug candidates [30]. Many developed drugs have not shown the positive therapeutic effects on AD patients and even can cause severe adverse reactions, such as meningitis [31] and cognitive impairment [32]. Recent animal and clinical studies have reported that Tau pathology can spread in the brain and cause cognitive impairment without the accumulation of Aβ, which may be due to the speeded clearance rate of Aβ in the brain upon anti-Aβ treatment for AD [33]. In addition, some non-amyloid treatment methods, such as circadian rhythm intervention [34], have gradually gained the attention by scholars. Recently, several new treatment proposals after summarizing a large number of clinical trial data have been put forward: including developing drugs to simultaneously or continuously target Aβ and Tau protein; non-biological targeted treatment strategies; or selecting mild cognitive impairment (MCI) patients or early AD patients as clinical trial subjects [35]. When APP is subjected to the cleavage by α-secretase and β-secretase, the β-carboxyl terminus of APP is released, and the Aβ48 and Aβ49 are produced through the endo-proteolytic(ε) cleavage by γ-secretase. Then, Aβ48 and Aβ49 can be γ-cleaved in the order of every three amino acid residues to generate shorter Aβ peptides, such as Aβ40 and Aβ37 [36]. The contents of these smaller Aβ peptides can be important indicators for evaluating γ-secretase activity. Among them, Aβ40 and Aβ42, mainly accumulated in nerve cells, are the most abundant and exhibit the substantial neurotoxicity [37]. Therefore, they are often used as biomarkers to predict mild cognitive impairment and AD. However, the Aβ37/Aβ42 ratio in CSF is more accurate in diagnosing AD because the change in Aβ37 level can better reflect the functional status of γ-secretase than Aβ40 [38]. Recently, based on the linear causal relationship in the amyloid cascade hypothesis, a new probabilistic model of A(Aβ)-T(Tau)-N(Neurodegeneration) has been proposed. Compared with the traditional hypothetical model, this model can be applied to different clinical types of AD [39]. However, recent animal or clinical studies have found that APP [40] or Aβ [41] in the brain does not always seem to be harmful. Only 30-40% of people with Aβ deposition in the brain will develop AD after the age of 70 years old, while approximately 50% of the populations never experience a cognitive decline for whole life [42, 43]. In recent years, when many scholars are conducting clinical trials of Aβ antibodies, they have found that many subjects have significantly reduced Aβ deposition in the brain. However, cognitive function has not been improved [44]. Similarly, neurodegeneration in the brain is persistent after APP is knocked out in animal models [45]. After analyzing brain imaging of 2,700 ordinary people, patients with mild cognitive impairment and AD patients at different periods, the appearance of AD symptoms may be caused by the reduction of soluble Aβ42 [41].

Interestingly, it is found that dense-core plaques are formed after activated microglia phagocytosed loosely organized Aβ plaques to relieve inflammation, thereby inducing the protective effects [46]. In addition, cognitive impairment is likely due to functional and structural impairment of neurons caused by hyperphosphorylation of Tau protein rather than Aβ [47], which reminds us that there may be a greater need to explore the difference with amyloid cascade hypothesis of AD. With the in-depth exploration of AD, new concepts and perspectives on the pathogenesis of AD continue to emerge, which gradually enriches the theoretical references of AD, and will be helpful for developing effective prevention and treatment strategies for AD [48].

## Inflammation and AD

**Fig. 1:**
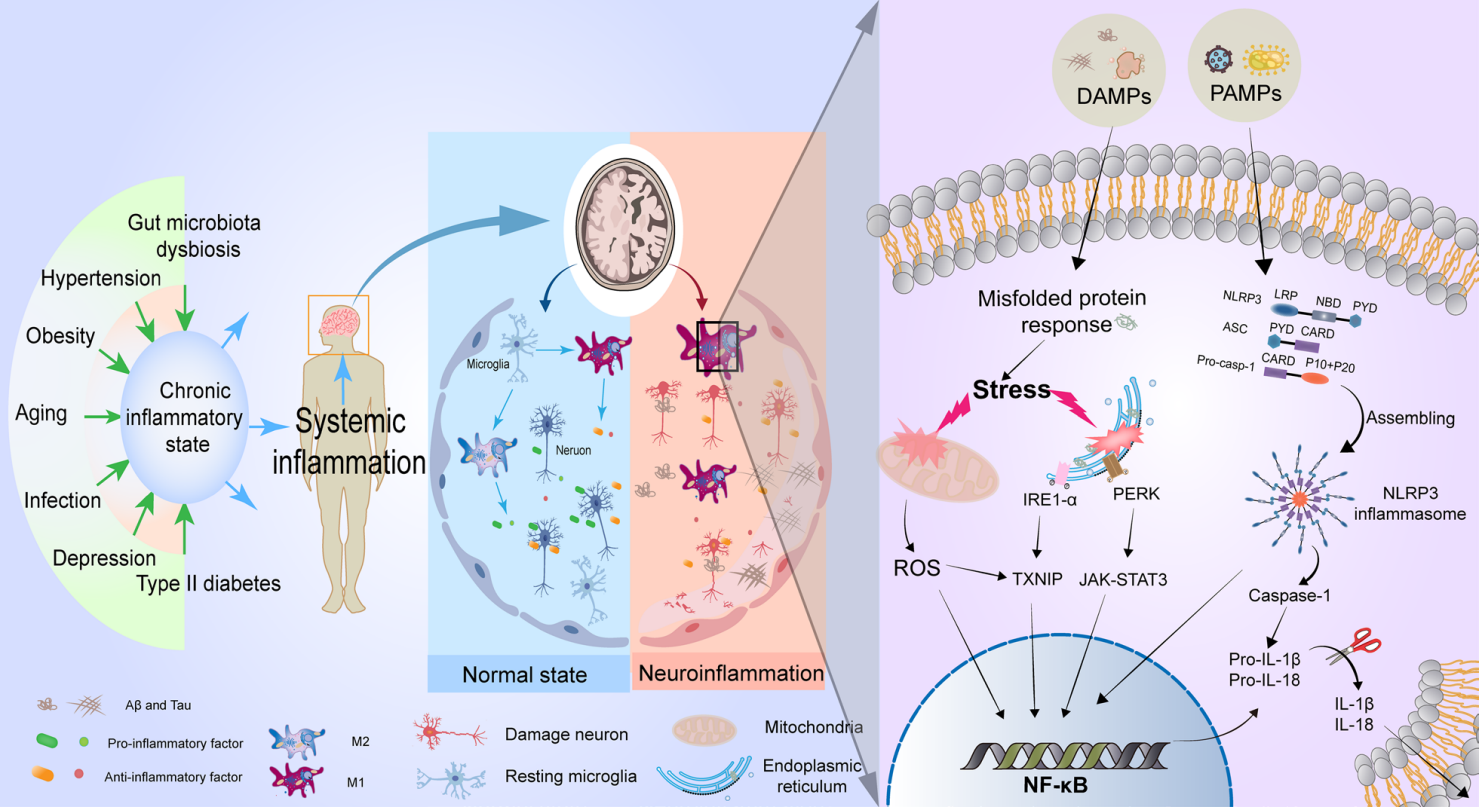
The molecular regulation of neuroinflammation for AD. At the early stage of AD, the body presents a chronic low-inflammatory state induced by aging, hypertension, type II diabetes, obesity, infection, and other risk factors, which can release a variety of danger-associated molecular patterns (DAMPs) or pathogen-associated molecular patterns (PAMPs) to activate immune responses of the nervous system. Resting microglia can be converted into a pro-inflammatory M1 phenotype to clear these danger signals for returning to a resting state. With the progression of the disease, under the continuous stimulation of DAMPs dominated by hyperphosphorylated Tau protein, extensive endoplasmic reticulum stress, oxidative stress, and the formation of NOD-like receptor protein 3 (NLRP3) inflammatory cells after assembly by NLRP3 in activated microglia are triggered, which promotes the entry of nuclear factor kappa-B (NF-κB) into the nucleus, thus resulting in the up-regulation of inflammatory genes (such as IL-1β and IL-18) to restore neural homeostasis. Thus, the accumulation of mis-folded proteins, M1-phenotype microglia, and inflammatory factors contribute to the neuroinflammatory microenvironment. Neuroinflammation also promotes the diffusion of hyperphosphorylated Tau protein in the brain, thereby creating a positive feedback loop to drive AD

According to the epidemiology, AD is mainly divided into two categories: early-onset familial AD (EOAD) and late-onset AD (LOAD), but their pathophysiology is very similar. From a genetic point of view, the pathogenesis of EOAD is closely related to the mutation of Aβ-related genes APP, presenilin-1 and presenilin-2. However, most AD cases (> 95%) belong to LOAD, whose most vital genetic risk factor is apolipoprotein E ε4 (APOEε4) [5]. In APOEε4 heterozygotes, the risk of AD is approximately 400%, when compared with approximately 1500% in homozygotes [49]. Dementia patients typically experience four stages of health, prodromal AD, mild cognitive impairment, and AD [50]. Prodromal AD refers to the stage from the first neuropathological changes in the brain to the first symptoms of AD. Diagnosis in the preclinical stage requires the absence of clinical signs and symptoms of AD and the presence of at least one biomarker of AD, which often requires early biomarkers for diagnosis [51, 52]. Long-term clinical cohort studies have documented the importance of platelet Tau variants as early diagnosis or the prevention of neurodegenerative diseases including AD [53, 54], which is consistent with relevant results, indicating that phosphorylated Tau in plasma may be an essential biomarker in the early stage of AD [55, 56]. Mild cognitive impairment and AD are assessed according to the severity of cognitive impairment. According to the 2022 World Alzheimer's Disease Report, up to 40% of dementia are correlated with lifestyles, including work stress, growth environment, and living standards [57]. Changing these adverse factors can prevent and delay AD to a certain extent [50]. The most significant risk factor for LOAD is aging, and other factors include obesity, diabetes, and cardiovascular diseases. The common feature of these factors is systemic chronic low-level inflammatory response [58]. Multiple meta-analyses have documented the inflammatory responses to AD [59, 60], and more and more studies have validated that inflammatory diseases such as bacterial infection [61], oral infection [62], and imbalanced intestinal flora are closely related to AD [63]. With the extension of age, body function reveals the gradual decline, and the integrity and permeability of the blood-brain barrier (BBB) to protect the brain from peripheral immunity are destroyed. The damaged BBB allows peripheral hormones, bacterial metabolites, and immunoglobulins to enter the brain to activate microglia, thereby activating the central immune system [58, 64, 65]. The levels of CD45 lymphocytes, interleukin-17 (IL-17), interferon-G (IFN-G), and interleukin-6 (IL-6) in CSF and blood of patients with AD are significantly increased [66], suggesting that activated immune cells can also participate in the immune response by entering the brain from the periphery. The presence of many reactive hyperplasia of microglia and astrocytes around SPs imply that the immune system is an essential event in the pathogenesis of AD. Observational studies have found that elevated glial fibrillary acidic protein (GFAP) and triggering receptor expressed on myeloid cell 2 (TREM2) in blood and CSF can serve as biomarker events for the diagnosis of early AD [67, 68]. TREM2 levels in CSF reveal the reduction before AD and the increase in early AD, and the final decrease again in late AD [69]. Plasma GFAP can more accurately reflect the changes in Aβ burden (not Tau protein) and disease severity in pre-symptomatic AD when compared with GFAP and TREM2 in CSF [70–72]. At the same time, TREM2 levels in CSF are closely related to Tau protein [73]. In addition, a biomarker analysis of healthy elderly, MCI patients, and AD patients has found that MCI patients with low levels of TREM2 in CSF or high levels of plasma TREM2 are more likely to accelerate the progression of AD [71]. In AD and other neurodegenerative diseases, the function of TREM2-mediated activation of microglia depends on the stage of disease progression and the type of microglia [74]. After summarizing the recent studies, relevant scholars propose that neuroinflammation is more than an incidental phenomenon of AD pathology. Conversely, neuroinflammation may help to drive the pathogenesis of AD. Epidemiological studies have confirmed that long-term users of anti-inflammatory drugs are less likely to suffer from AD [75]. In addition, according to the neuroinflammation hypothesis, new treatments for the alleviation of AD using natural anti-inflammatory products such as curcumin are gaining attention [76–78]. Therefore, AD has regarded as one of the secondary neurological diseases of chronic inflammation [79] (Fig. 1).

### Pathogenesis of neuroinflammation in AD

Neuroinflammation has been first proposed as the essential inducer of AD. Long-term stimulation of "damaged signals" can activate the innate immune system and trigger a series of inflammatory cascades, which is closely related to neurodegeneration in AD [80–82]. Relevant studies have pointed out that the importance of Tau in AD neuroinflammation may be far greater than that of Aβ [81]. Furthermore, the neuroinflammation hypothesis also seems to hold for other neurodegenerative diseases with tauopathy [83]. The body is exposed to various stimuli in daily life, including exogenous PAMPs and in vivo DAMPs, such as bacterial endotoxins and mis-folded proteins. As a defense mechanism, neuroinflammation protects the brain mainly by clearing DAMPs or PAMPs.

**Fig. 2:**
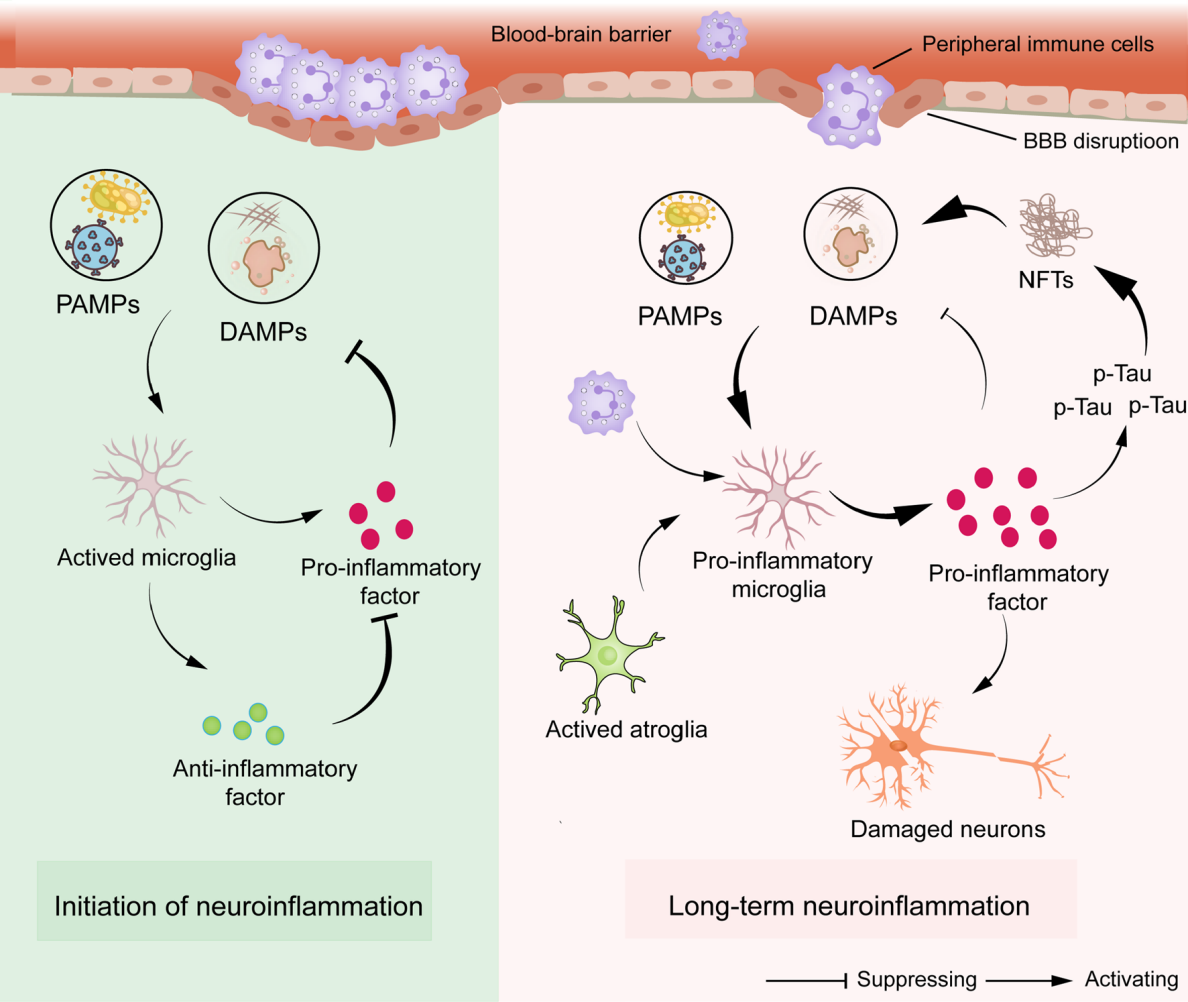
The pathogenesis and process of neuroinflammation in AD. After damaged signals (DAMPs and PAMPs) invade the brain, microglia become pro-inflammatory cells and secrete inflammatory factors to clear these signals. Subsequently, anti-inflammatory microglia emerge and secrete anti-inflammatory factors to counteract inflammatory factors. Damage signals in AD cannot be entirely cleared by microglia, so pro-inflammatory microglia keep secreting inflammatory factors to damage neurons. Inflammatory factors continuously intensify the phosphorylation of Tau protein and the formation of NFTs, which are the major substances of damaged signals in AD, finally forming a vicious circle

PAMPs and DAMPs play a protective role by binding to pattern recognition receptors (PRRs) to initiate the activation of microglia and promote the release of mature pro-inflammatory cytokines from microglia to clear DAMPs and PAMPs. However, dysregulated DAMPs and PAMPs can pathologically exacerbate the levels of chronic inflammation with the extension of age [84]. When these stimuli are chronically present, microglia remain activated and become a long-term source of inflammatory factors [85]. However, long-term inflammatory stimulation may show a wrong side [86]. Inflammatory factors can also activate protein-related kinases, such as cyclin-dependent kinase 5, to promote the formation of NFTs from hyperphosphorylated Tau protein and further aggravate the inflammatory response in the brain [87–89]. Blocking the formation of inflammasomes in microglia can reduce neuroinflammation and delay the pathogenesis of AD [90]. On the one hand, communication between microglia and astrocytes plays a vital role in neuroinflammation [91]. As the most numerous glial cells in the brain, astrocytes regulate blood flow, maintain the blood-brain barrier, and maintain a stable environment for synapses and neurotransmitters [92]. The switch of both glial cells between pro-inflammatory and anti-inflammatory phenotypes promotes the transformation of each other. For example, IL-1α, TNF-α, and complement secreted by activated microglia can convert astrocytes to a pro-inflammatory phenotype [93]. Interestingly, neuroinflammation due to the activation of astrocytes is responsible for the degenerative development of tauopathies [94]. Recent animal and clinical studies have shown that a subset of astrocytes can influence microglial function by releasing IL-3, thereby allowing microglia to focus on clearing phosphorylated Tau and NFTs without destroying neurons [95]. In addition, the primary source of the protective IL-3 is the brain, and its expression is reduced in the brain of 5xFAD mice [95]. On the other hand, both high-risk factors for AD (aging and APOE4) and astrocyte damage disrupt the integrity of the blood-brain barrier, thus allowing the immune privilege in the brain to be disrupted [96]. Blood-borne DAMPs and PAMPs, such as complement, monocytes, and gut microbiota metabolites, enter the compromised blood-brain barrier to promote specific immune responses and exacerbate the burden on the innate system, thereby accelerating neurodegeneration [97]. In addition to the activation of peripheral T cells, the number of CD8^+^ T cells in the CSF of MCI and AD patients is significantly higher than that of normal subjects [98]. The experimental results of AD model animals have also confirmed that the neuroinflammatory events of AD include not only the activation of immune cells, but also the infiltration of activated peripheral immune cells in the brain [97]. Peripheral immune cells can penetrate brain tissue and bind to glial cells, thus reducing AD pathology and cognitive impairment in AD-transgenic mice [99, 100]. However, the role of peripherally derived immune cells remains controversial. In brain tissues of AD patients, the level of CD3^+^ T cells in peripheral blood vessels is related to Tau protein, rather than Aβ [101], suggesting that Tau-related neurodegenerative changes drive the intervention of peripheral immune substances. Thus, the interaction between microglia and astrocytes and the disruption of brain immune privilege plays essential roles in neuroinflammation (Fig. 2).

### Neuroinflammation as an upstream core event in AD pathogenesis

The immune system of the brain and the levels of inflammatory biomarkers in blood largely contribute to the occurrence and development of AD due to hyperphosphorylated Tau protein accumulation, neuronal damage and cognitive impairment [102]. In a study with CSF examination and brain magnetic resonance images of 300 people over the age of 60 years old, it is found that even people without dementia symptoms exhibit a trend of significantly higher inflammatory biomarkers [103, 104]. When lipopolysaccharide (LPS) induces a chronic inflammatory state in mice, AD-related pathological processes in brain tissues of mice are significantly aggravated [105]. These experimental data in animal models and humans suggest that neuroinflammation is an upstream event in AD pathogenesis. Large-scale samples also show the increased C-reactive protein (CRP) in the blood of the people at middle age, which is similar with AD patients [106]. However, some clinical studies have also reported that lower plasma CRP levels are more prone to AD [107]. It is worth noting that CRP elevation is an acute inflammation as the feedback adaptation, indicating that chronic inflammation may be an essential factor for inducing AD. In addition, neuroinflammation can induce M1-phenotype microglia to release pro-inflammatory factors, whose complements modulate neuronal and synaptic damage, thereby resulting in the spread of Tau protein from the medulla or pons to the entire cortex [108]. In animal experiments, sustained expression of interleukin-1 beta (IL-1β) in the hippocampus can activate astrocytes and microglia to trigger a robust inflammatory response, ultimately leading to memory impairment [109]. For AD patients with long-term application of NSAIDs, symptomatic or asymptomatic progression of AD is significantly slowed down [78]. Therefore, neuroinflammation may be the upstream core event with the function of triggering AD.

### Critical features of neuroinflammation in AD

Microglial activation has been recognized as a critical hallmark event in the pathogenesis of AD [110]. The activation process of microglia is closely related to the release of endocannabinoids. Microglia are the primary source of endocannabinoids under physiological conditions, but the expression of cannabinoid receptors in resting microglia is low [111]. Under normal conditions, microglia play a vital role in removing the aggregation of mis-folded proteins or forming a glial barrier to prevent mis-folded proteins from accumulation and spreading [112]. Activated microglia can also express cannabinoid receptors to activate endogenous cannabinoid signaling to regulate the polarization and proliferation of microglia, ultimately reducing the inflammatory response [113, 114]. According to the microglial dysfunction hypothesis, when the damage factors exist for a long time, the chronically activated microglia will suffer from malnutrition and apoptosis, so they cannot perform their average clearance and monitoring functions, eventually accelerating the formation of neuroinflammation and neuronal degeneration [115–117]. Using the tracking technology of microglia, positron emission tomography (PET) data show that microglial activation is observed in the elderly and patients with MCI. Moreover, AD patients show a sequential increase in activated microglia, and this phenomenon may be positively correlated with the level of phosphorylated Tau protein in the brain [108], which is also consistent with the results from RNA-seq analysis of brain tissue samples from AD patients [118]. Moreover, chronically activated microglia-mediated inflammatory events may be also related to the maturation of precursor interleukins induced by Aβ-activated NLRP3 in microglia [119]. The initiation signals of NLRP3 include several internal and external activators, such as PAMPs and DAMPs. Mis-folded proteins are the most typical DAMPs in AD. In APP/PS1, APP/PS1/NLRP3^−/−^, and APP/PS1/Caspase-1^−/−^ model mice, reduced NLRP3 and Caspase-1 activity can promote the clearance of DAMPs and restore learning and memory capacity of the mice [90]. Interestingly, in APP/PS1/NLRP3^−/−^ mice, M2 phenotype microglia are the majority, indicating that the NLRP3-Caspase-1 axis plays a vital role in the pathological process of AD, and this axis may be a potential therapeutic target through suppressing neuroinflammation. At the same time, the increased expression of Caspase-1 in brain tissues of MCI and AD patients is determined [90], implying that the NLRP3 inflammasome is at a chronic activation state during the pathogenesis of AD. Indeed, apoptosis-associated speck-like protein containing caspase recruitment domain (CARD) (ASC) specks in the inflammasomes are released into the intercellular space. Adjacent microglia can take up these specks, thus leading to the spreading of inflammatory factors in the brain and long-term activation of the immune system. However, neurodegeneration is ameliorated after applying the ASC speck antibody [120–122]. High expression of NLRP3 and Caspase-1 can suppress the function of microglia and then accelerate the pathological process of AD [123, 124]. Notably, the changes in inflammatory responses appear to be triggered by Tau pathology alone. Previous experiments have shown that the inflammatory response in the AD brain is pronounced in the presence of Tau pathology only, with the essential role in the involvement of microglia [125, 126]. Furthermore, in AD, Tau protein propagates itself into the brain via the NLRP3-ASC signal pathway, so the aggregation of Tau protein appears to be more inflammation than Aβ and is associated with the progression of AD symptoms [127], indicating that Tau protein is likely to be the dominant player in neuroinflammation. In in vitro studies, microglial activation may lead to pathological aggravation of Tau proteins again, which supports this positive feedback mechanism and explains the rapid progression of cortical NFTs in AD [128]. Consequently, reducing the expression of these inflammatory factors and the activation of NLRP3 in microglia are helpful for alleviating cognitive impairment of AD mice [129]. The inhibition of NF-κB and NLRP3/Caspase-1 signal pathways in microglia is a potential therapeutic strategy for AD [130]. Therefore, chronic activation of microglia and NLRP3 are the significant features of neuroinflammation in AD.

### Regulatory mechanisms of neuroinflammation in AD

Long-term endoplasmic reticulum dysfunction is closely related to cognitive impairment and memory loss in AD [131]. In the AD brain, the continuous accumulation of Aβ and hyperphosphorylated Tau leads to the continuous increase of endoplasmic reticulum stress (ERS), which sequentially activates the unfolded protein response (URP) and NF-κB, through the PKR-like ER kinase (PERK)/JAK1/STAT3 and inositol-requiring enzyme 1 (IRE1)/thioredoxin-interacting protein (TXNIP) signal pathways, thereby causing aseptic inflammatory responses [132, 133]. The significant elevation of URP markers such as immunoglobulin heavy-chain-binding protein (BiP) is detected in the hippocampus of AD patients, especially in neurons of the CA1 and CA2 regions [134]. Moreover, ERS and hyperphosphorylation of Tau protein can mutually induce and promote each other, thereby exacerbating the pathogenesis of AD [135]. In addition to participating the process of relieving ERS, URP itself can activate inflammatory pathways in immune responses, including NF-κB, mitogen-activated protein kinase (MAPK) family protein c-Jun N-terminal kinase (JNK), and p38 [136]. It has been reported that ERS induced by Aβ and NFTs in the hippocampus of 5xFAD mice could also up-regulate TXNIP, although it does not affect the expression level of its negative regulator or redox regulator thioredoxin (TRX) [137]. ERS also can lead to increased expression and secretion of IL-1β in the hippocampus via the activation of TXNIP/NLPR3 signal pathway in the brain [138], which is consistent with the previous report that ERS can induce inflammation in diabetes to modulate islet β cell death [139]. The expression of cytokines and chemokines is enhanced by the activated PERK signaling, and conditional knockout of PERK can enhance synaptic plasticity and memory function in APP/PS1 transgenic mice [140, 141].

On the other hand, oxidative stress is a proximal event in AD pathogenesis prior to AD symptoms [142]. In SAMP8 mice with significant oxidative stress, the cognitive function of the mice can be alleviated or even reversed after the application of antioxidants, Aβ antibodies, and APP antibodies, respectively [143, 144]. Similarly, enhancing antioxidant capacity can suppress the expression of Aβ and APP and ultimately restore the impaired memory capacity of 3xTG transgenic mice. At the same time, the reduction of superoxidase dismutase (SOD) activity in the cytoplasm can lead to the formation of more Aβ oligomers [145, 146]. Oxidative damage can disrupt oxidative homeostasis, thus resulting in the production of reactive oxygen species (ROS) [147]. In the cerebral cortex of APP/PS1 transgenic mice at the age of 7, 12 and 20 months, the ratio of NAD^+^/NADH is decreased when compared with that of wild-type mice, and the inflammatory signal pathway in the hippocampal tissues is decreased upon the treatment with NAD^+^ supplementation nicotinamide riboside (NR), as shown in reducing neuroinflammation and alleviating cellular senescence through the cGAS-STING signal pathway [148]. Interestingly, TXNIP is not only implicated in ERS, but also acts as an endogenous inhibitor of the antioxidant TRX [149]. TRX is a major intracellular thiol-reducing and ROS-scavenging protein, and the binding of TXNIP to TRX can inhibit TRX activation and trigger oxidative stress. In AD, the antioxidant effect can reveal a decreasing trend with the decrease of nuclear factor-related factor 2 (Nrf2) level [150], as confirmed that Dl-3-n-butylphthalide (NBP) inhibits NLRP3 inflammasomes and delays the pathological process of AD through the Nrf2-TXNIP-TRX signal pathway [151, 152]. Therefore, ERS and oxidative stress, at the early stage of AD, can execute the protective mechanism and the suppression of neuroinflammation in AD.

## Exercise and inflammation

Today, it is widely accepted that physical activity is essential for maintaining and promoting health. In 2020, the World Health Organization (WHO) proposed that all adults should have 150–300 min of moderate-intensity physical activity or 75–150 min of vigorous-intensity physical activity per week [153]. Exercise has a wide range of effects on the immune system. Exercise can increase the inflammatory state in the body by promoting the hypothalamus-pituitary-adrenal axis, improving cell survival environment and anti-apoptosis, optimizing the functional status of autophagy, and regulating endocrine. Exercise can also trigger an inflammatory response, thus releasing ROS and reactive nitrogen species (RNS) by damaging muscle, suppressing immune systems, activating inflammation, and depleting glycogen [23, 154]. According to the “open-window” hypothesis, a compromised immune system after strenuous exercise increases the risk of contracting an upper respiratory tract infection [155]. However, strenuous exercise also increases immune activity by redistributing immune cells to desired tissues, thereby reducing the chance of infection [156]. The effect of exercise on inflammation is related to the type, intensity, duration of exercise training, and individual or tissue differences [157]. For example, regular moderate-intensity physical activity can promote an anti-inflammatory state, but high-intensity physical activity or competition has been shown to activate the inflammatory response [158]. An acute exercise results in peak inflammation in muscle tissue within the first few hours [159], and this initial pro-inflammatory response is quickly counteracted by anti-inflammatory effects after regular exercise [160]. In addition, studies have shown that exercise intensity can be adjusted by assessing the level of neopterin, an endogenous immune activation marker, to determine the level of inflammation in the body during or after exercise [157]. In an animal model of LPS-induced inflammation, 3-week moderate-intensity treadmill exercise reduces neopterin levels and suppresses immune-inflammatory responses [161]. Regular moderate-intensity physical activity is thought to have immunomodulatory effects, enhancing defenses against infection and reducing the incidence of chronic diseases [155]. Therefore, the anti-inflammatory effects of exercise are more likely to be triggered by long-term moderate-intensity physical activity.

Increasing animal and clinical studies show that scientific and reasonable exercise can stimulate the body to produce an anti-inflammatory phenotype state [162]. In addition to enhancing memory and cognitive capacity, regular aerobic exercise can also reduce the levels of CRP, IL-6, tumor necrosis factor alpha (TNF-α), soluble tumor necrosis factor receptor-1 (sTNFR1), and soluble tumor necrosis factor receptor-2 (sTNFR2) and promote the production of anti-inflammatory factors such as interleukin 10 (IL-10), IL-1 receptor antagonist (IL-1RA), interleukin 4 (IL-4) and transforming growth factor beta-1 (TGF-β1) [163, 164]. For example, regular aerobic exercise of patients with metabolic syndromes can reduce IL-6 by 30%, TNF-α by 15% and leukocyte counts by 15% in blood [165]. Not only that, regular physical activity also can exert an anti-inflammatory effect on chronic and inflammatory diseases. Similarly, weekly moderate-intensity aerobic exercise can be helpful to reduce peripheral inflammation levels of the people with type 2 diabetes [166]. Regular physical activity can promote the tropism of neutrophils and natural killer (NK) cells to optimize their functional status [167]. Similarly, the elderly at the average age of 71 years old reveal a threefold decrease in the number of pro-inflammatory monocytes CD14 and CD16 in their blood after strength training of legs and chest [168]. In addition, regular exercise (aerobic and resistance exercise) can also reduce the secretion of pro-inflammatory cytokines in young people and induce skeletal muscle to release anti-inflammatory mediators such as IL-6 [169]. Exercise-induced increase of circulating IL-6 and increased plasma levels of anti-inflammatory factors, such as IL-1RA and IL-10. IL-1RA can inhibit IL-1β signaling, while IL-10 can inhibit the production of inflammatory factors, including TNF-α [170]. Notably, the expression of toll-like receptors on the monocyte membrane is decreased after an acute prolonged exercise, thereby affecting the secretion of pro-inflammatory factors [164]. In addition, prolonged exercise can also affect the number of different T cells including regulatory T cells for influencing the immune system [171]. Therefore, exercise may be one of the crucial ways to regulate immune and inflammatory responses.

## Exercise and AD

Since current drugs for AD have not achieved good clinical efficacy, people have focused their attention on changing lifestyles for the prevention and treatment of AD. The 2022 report on AD lists high-risk factors for AD at all stages. For example, one of early AD risk factors includes poor education; late risk factors are smoking, physical inactivity, depression, social isolation, diabetes, and air pollution. In addition, 40% of AD patients can be prevented or delayed by modulating these controllable risk factors [172]. Several decades observational studies have confirmed this fact [173, 174]. Therefore, the study recommends the prevention of dementia based on the entire life cycle, such as regular physical activity in middle and old age [175]. It is well known that a sedentary lifestyle is associated with impaired cognitive function in AD populations, so one possible approach to ameliorating AD is regular physical activity [176]. A recent retrospective analysis including 160,000 participants has found that participants with regular and active exercise have a lower risk of developing AD by 45% [177], suggesting that physical activity has a more positive role in preventing AD, and similar results are also reported in other literature [178]. Comparable results are also achieved in a prospective study of 716 elderly subjects [179]. In addition, exercise positively affects high-risk factors for AD, including hypertension, type II diabetes, obesity, and hyperlipidemia [180]. On the other hand, regular physical activity has been reported to have multiple benefits in relieving AD symptoms in both human and animal experiments [181]. For example, physical activity has improved learning and memory capacity by increasing long-term potentiation (LTP) and neurogenesis [182], suggesting that physical activity may also be associated with structural and functional changes in the brain. The 5-month voluntary wheel running can reduce Aβ40 and Aβ42 levels in the brains of 3xTG transgenic mice [183]. Similarly, 3-month-old APP/PS1 transgenic mice reveal the significantly reduced Aβ accumulation in the brain after five months of treadmill running [184]. In addition to reducing the expression level of β-secretase in transgenic mice, exercise can also change the activity of γ-secretase [185] and promote the activity of α-secretase in a Sirt1-dependent manner [186]. Furthermore, exercise also can promote lactate secretion in skeletal muscle in a Sirt1-dependent manner to increase the level of brain-derived neurotrophic factor (BDNF) in the hippocampus for enhancing neuronal function and ultimately restoring the memory capacity of mice. At the same time, BDNF can reduce β-site amyloid precursor protein cleaving enzyme 1 (BACE1) activity [187, 188]. The capability of the brain to synthesize BDNF reveals an increase by approximately 2–3 folds during prolonged exercise [189]. Therefore, exercise may reduce the content of Aβ in AD model mice by regulating the activities of α-, β-, and γ-secretase. Accumulating evidence suggests that microglia play essential roles in many aspects including the regulation of Aβ, hyperphosphorylated Tau, neuronal function, and synaptic plasticity [190]. For example, microglia can promote the phagocytosis capacity of Aβ [191]. Astrocytes can promote the positional change of the aquaporin 4 (AQP4) to increase the clearance rate of Aβ. Similarly, a transient and prominent microglial activation state in the hippocampus of 3xTG transgenic mice after 3 weeks of voluntary wheel running is also observed [192]. Reducing the deposition of Aβ in the hippocampus can increase learning and memory capacity of mice. Therefore, the effects of exercise on preventing and delaying AD are multifaceted. After 6-month resistance exercise in 100 MCI patients aged 55–85 years old, their memory, attention, and executive skills are improved significantly during and 12 months after exercise [193]. In terms of different AD patients, MCI patients, or healthy people, exercise is effective against high-risk factors for AD. It also can enhance the resilience against AD, thereby improving cognitive reserve function and brain tissue plasticity [194]. However, some human experiments have shown that 16-week aerobic exercise does not reduce the content of Aβ in the brain of AD patients [195]. This result may suggest that the anti- and pro-inflammatory effects of exercise may depend on various factors such as exercise intensity and duration.

## Exercise suppresses neuroinflammation for ameliorating AD

**Fig. 3:**
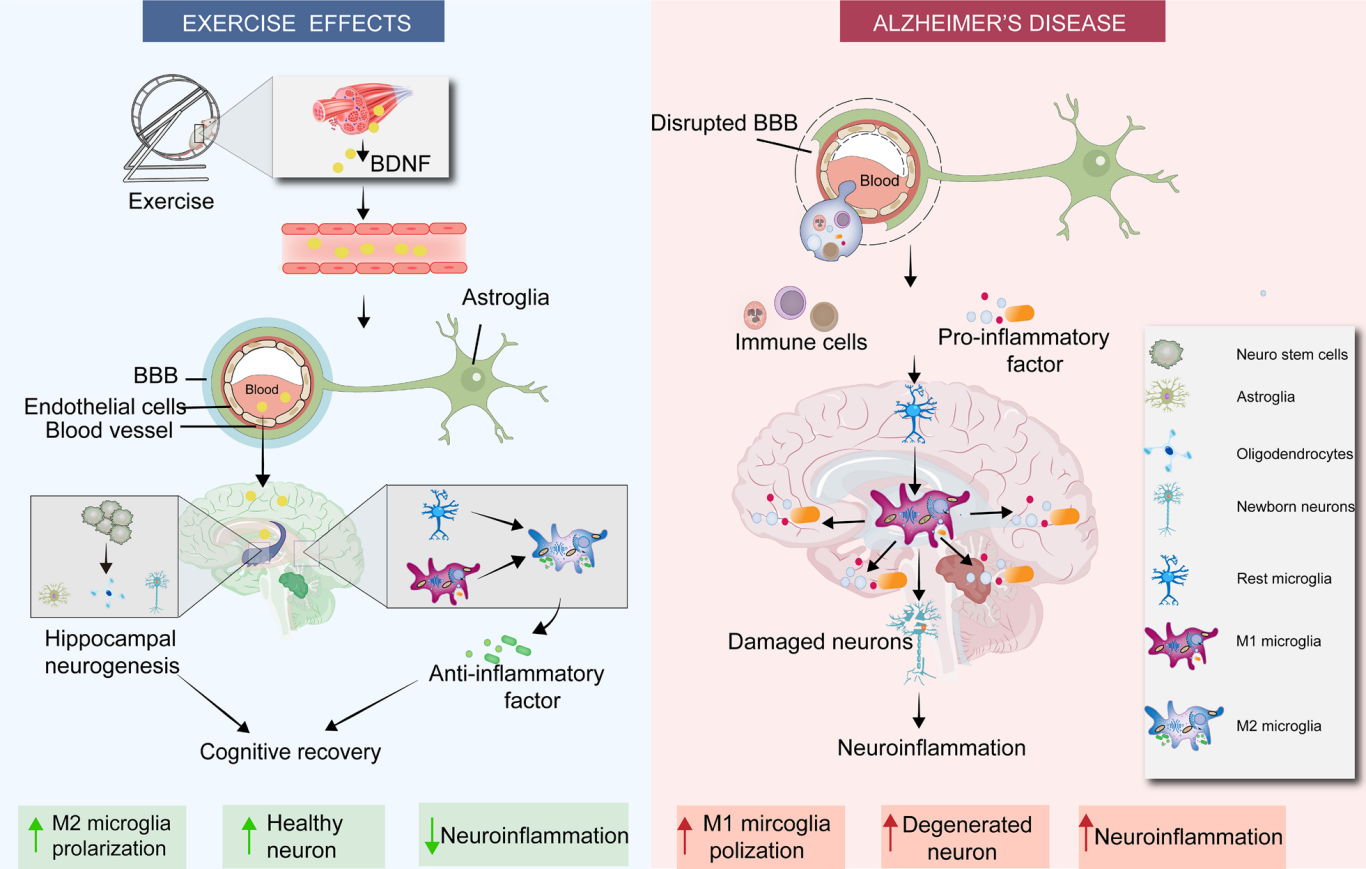
Exercise modulates neuroinflammatory responses to ameliorate AD. First, exercise suppresses chronic inflammation in the body through reducing circulating levels of pro-inflammatory factors and immune cells. Second, exercise restores the permeability and integrity of the BBB by repairing damaged endothelial cells and tight junctions, ultimately preventing inflammatory factors and immune cells from entering the brain. Third, exercise inhibits the pro-inflammatory M1 phenotype and stimulates the anti-inflammatory M2 phenotype to increase the levels of anti-inflammatory factors in the brain, thereby restoring homeostasis. Finally, exercise triggers adult hippocampal neurogenesis (AHN) by inducing the expression of BDNF in the brain and muscle, thus leading to the continuous formation of new neurons, astrocytes, and oligodendrocytes. These new cells can replace the corresponding senescent and damaged cells, thus remodeling the high-loading state caused by neuroinflammation. Therefore, exercise inhibits the neuroinflammatory response through four effects and ultimately delays the pathological process of AD and alleviates symptoms

AD has been recognized as a neurodegenerative disease caused by a chronic inflammatory response [196]. The aging of organisms, the accumulation of progressive damage, and the loss of the reserve function of each organ may be related to the inflammatory response [197]. Long-term high-frequency physical exercise can alleviate the degeneration of the body system for delaying aging and improving physical fitness [198]. In studies on naturally aging animals and healthy people with different age, it is found that lifelong exercise can effectively alleviate the systemic inflammatory response in mice by inhibiting the levels of pro-inflammatory factors and increasing the levels of anti-inflammatory factors, respectively [199, 200]. A recent review proposes that the anti-inflammatory properties of exercise can suppress the inflammatory state of AD and ameliorate the pathophysiological characteristics of AD [201]. Numerous studies have shown the decreased immune responses and ameliorated cognitive impairment in elderly, and MCI and AD patients after a period of physical activity [202]. The studies on exercise-mediated neuroinflammation in AD models are listed in Table 1. Non-work-related exercise has a specific protective effect on AD. For example, exercise can improve the judgment and problem-solving capacity of AD patients and modulate serum inflammatory markers in AD patients [203]. Similarly, human and animal data show that exercise can increase TREM2 levels in CSF of AD patients, maintain plasma TREM2 levels in APP/PS1 mice, and reduce plasma GFAP levels in multiple sclerosis patients [204–206]. The plasma of long-term exerciser has been shown to suppress inflammatory responses in the hippocampus by inhibiting complement-related signal pathways and preventing neuroinflammation [207]. Although these causal mechanisms are still under debate, the anti-inflammatory effects of exercise are meaningful and feasible as a therapeutic strategy for aging-related neurodegenerative diseases. Therefore, the inhibition of inflammatory responses may be a potential target for ameliorating AD (Fig. 3).

**Table 1:** Exercise suppresses neuroinflammation in AD

Model	Exercise mode	Exercise duration and frequency	Changes in inflammatory markers	Changes in other indicators	Source of inflammatory markers	References
AD rats (male)	Swimming	30 min/time; 7 times/week; 4 weeks	IL-10 ↑; IL-6 ↓	Aβ, Tau ↓; BDNF ↑	Serum	[270]
SD rats (2.5 months old, female)	Swimming	60 min/time; 7 times/week; 4 weeks	IL-10 ↑; TNF-α, IL-1β, IL-6 and IL-18 ↓	Aβ, Tau ↓; Memory impairment rescuing	Hippocampal tissue	[271]
TG2576 mice (17–18 months old)	Voluntary wheel running	3 weeks	IL-1β, TNF-α ↓; IFN-γ, MIP-1α ↑; CD40 ↑	Aβ ↓	Hippocampus and cortex	[272]
5xFAD mice (2–3 months old, female)	Voluntary wheel running	24 weeks	IL-1β →	Cognitive function → ; Anxious behavior↑	Hippocampal tissue	[267]
BALB/C mice (4 and 22 months old, Male and female)	Voluntary wheel running	10 weeks	Expression levels of MHCII and CD86 ↓	–	Hippocampal tissue	[273]
APP/PS1 mice (17 months old)	Voluntary wheel running	3 weeks	Expression of inflammatory genes ↓	Proliferation of precursor neurons and glial cells ↑	Plasma	[207]
3xTG mice (9 months old, male)	Resistance exercise	3 times/week; 4 weeks	TNF-α, IL-1β, and IL-1β ↓; IL-10 and IL-6 →	Aβ, p-Tau ↓; Activation of glial cells ↓; Memory impairment rescuing	Serum, frontal cortex, and hippocampus	[274]
APP/PS1 mice (6–7 months old, Male)	Resistance exercise	5 times/week; 4 weeks	IL-1α, IL-6 and IL-4 ↓	Number of microglia ↑; Aβ plaques in hippocampus ↓	Hippocampal tissue	[275]
Wistar rats (18 months old, Male)	Treadmill running	30 min/time; 1.4 weeks (10 days)	TNF-α, IL-1β and IL-6 ↓	–	Hippocampal tissue	[276]
3xTG mice (16 months old)	Treadmill running	60 min/time; 5 times/week; 12 weeks	TNF-α, IL-6, IL-1b, COX-2, and NK-κB ↓	Number of activated glial cells ↑	Hippocampal tissue	[277]
APP/PS1 mice (3 months old, male)	Treadmill running	45 min/time; 5 times/week; 12 weeks	TNF-α and IL-1β ↓; IL-10 and TGF-β ↑	Cognitive function ↑; Proportion of M2 phenotype microglia ↑	Hippocampal tissue	[213]
C57BL/6 mice (4 and 18 months old)	Treadmill running	50 min/time; 1.4 weeks (10 days)	IL-1β in hippocampus ↓	Proportion of M2 phenotype microglia ↑; Cognitive function ↑	Hippocampal tissue	[210]
Swiss mice (Male)	Treadmill running	40 min/time; 5 times/week; 4 weeks	IL-1α, NLRP3 and Caspase-1 expression ↓; IL-1β →	–	Hippocampal tissue	[217]
SD rats (Male)	Treadmill running	20 min/time; 5 times/week; 4 weeks	TNF-α and IL-1β ↓; IL-10 and IL-4 ↑	Aβ and p-Tau ↓; Proportion of M2 phenotype microglia ↑	Hippocampal tissue	[215]
211 elderlies (67 ± 5 years old)	Habitual physical activity	1 week	Neutrophils ↑; IL-6, IL-8, CRP, IL-10 and IL-13 →	–	Serum and plasma	[278]
368 seniors (70–89 years old)	Personalized exercise intervention	45 min/time; 3 times/week; 16 weeks	IL-8 ↓; IL-2, IL-6, IL -1Rα, IL-15 and TNF-α →	–	Blood	[279]
AD patients (Average 68.3 years old)	Exercise program (40–60% of heart rate reserve)	60 min/time; 2 times/week; 11 weeks	IL-4 ↑, IL-1β and TNF-α →	Cognitive function ↑; Spatial judgment ↑; Self-awareness ↑	Blood	[203]
Seniors (Average 78 years old)	Moderate-intensity aerobic exercise	45 min/time; 3 times/week; 16 weeks	CRP ↓	–	Blood	[280]
AD patients (Average 70.6 years old)	Moderate to high-intensity physical exercise	60 min/time; 3 times/week; 16 weeks	IL-6 ↑; TREM2↑, IL-10, IL-1β, IL-8, IL-13, and TNF-α →	–	Blood and CSF	[205]
AD patients (Average 73.05 years old)	Aerobic exercise	–	IL-6, IL-1β, and TNF-α↓	Cognitive function↑	Blood	[281]
AD patients (65–75 years old)	Aerobic exercise	45 min/time; 3 times/week; 8 weeks	TNF-α and IL-6↓	Quality of life↑	Blood	[282]
MCI patients (Average 65.72 years old)	Aerobic and Resistance exercise	40 min/time; 3 times/week; 16 weeks	IL-6, IL-8 and IL-1β → ; IL-15 and TNF-α↓	Cognitive function↑	Blood	[269]

Legend: ↑, level increase; ↓, level decrease, → , no significant change

### Neuroimmune-modifying effects upon exercise interventions

The innate immune system is the primary defense against exogenous pathogens and endogenous infections in the body. PAMPs or DAMPs bind to pattern recognition receptors (PPRs) on the membrane of glial cells to release cytokines, nitric oxide (NO), and other factors for defense [208]. In many animal models and humans with AD, astrocytes and microglia are significantly activated in areas with the initiation of AD pathology (frontal cortex and hippocampus) [209]. Activated microglia and astrocytes transform from a rest state to a pro-inflammatory state and play a protective role in clearing these damaged insults. However, under the long-term stimulation of injury factors, glial cells are continuously activated to stimulate the release of pro-inflammatory factors for restoring the cellular homeostasis in the body. As mentioned above, the activation state of microglia and corresponding phenotypic transformation play a key role in inflammatory responses in AD. After 10-day treadmill running of 4-month-old and 18-month-old C57BL/6 mice, exercise can promote the phagocytic capacity of mouse microglia, thereby reducing the content of IL-1β and reversing the decline of hippocampal neurons and memory capacity [210]. In multiple studies, the activation of microglia is suppressed in the hippocampus of APP/PS1 mice after 4 or 5 months of aerobic exercise [211, 212]. In addition, the treadmill running for 12 weeks promotes the transformation of microglia from the M1 phenotype to the M2 phenotype, thereby reducing inflammation and oxidative damage in hippocampal tissues, and finally resulting in the improvement of cognitive performance of mice [213].

Moreover, exercise can also inhibit the excessive activation of hippocampal microglia in aged mice, maintain the homeostasis of the nervous system, and effectively prevent neuronal damage in aged mice [214]. Furthermore, in streptozotocin (STZ)-induced diabetic model rats with AD-like symptoms, treadmill running increases the proportion of M2 phenotype microglia and attenuates oxidative stress-induced injury in the brain [215]. Therefore, exercise protects brain by suppressing the immune-inflammatory response by increasing the number and phagocytic capacity of M2 phenotype microglia, whose mechanism may be related to PPRs expressed on the microglial membrane. When the AIM2-like receptor 2 (ALR2) function is lost, it can induce the transformation of microglia from M1 phenotype to M2 phenotype and reduce the inflammatory response [216]. Exercise can up-regulate the expression of triggering receptor expressed on myeloid cell 2 (TREM2) and scavenger receptor A (SR-A) to enhance the neuroprotective function of microglia [205]. Moreover, a recent study has pointed out that four weeks of treadmill running can reduce NLRP3 content and Caspase-1 activity in the hippocampus of the mice after lateral ventricle injection of Aβ40 [217]. Numerous animal studies have also confirmed that inhibiting NLRP3 in microglia is a potential target for the treatment of AD in the future [218, 219], indicating that the regulation of PPRs in microglia by exercise is a potential immunotherapeutic direction. However, single-cell transcriptomics have revealed that microglia might have multiple phenotypes, including disease-associated microglia (DAM), and microglia with the coexistence of different phenotypes during the progression of AD [220, 221]. It is warranted in the future to investigate whether exercise has differential effects on these subdivided microglial phenotypes. Furthermore, whether exercise has a similar effect on astrocytes also needs to be explored [222].

On the other hand, exercise can reduce the impact of peripheral inflammatory factors on the central system. The BBB comprises endothelial cells, a basement membrane containing pericytes and astrocytes. Previous studies have confirmed that BBB is closely related to the occurrence and development of AD and is also a key point in preventing and treating AD [223]. Loss of BBB integrity allows cytokines and immune cells to enter the central nervous system (CNS), thereby activating glial cells and leading to changes in the extracellular milieu. Exercise can reduce TNF-α level in blood and enhance BBB function in patients with type II diabetes. Regular exercise training down-regulates pro-inflammatory cytokines, such as IL-6, and TNF-α, associated with low-grade systemic inflammation [224]. In AD, the accumulation of ROS activates metalloproteinases, thus leading to the disruption of BBB integrity [225]. BBB dysfunction during the progression of AD affects Aβ clearance and endothelial trafficking, impairs endothelial and pericyte function, disrupts tight junction (TJ) integrity, activates glial cells, and promotes leukocyte recruitment in the brain [226]. Physical activity inhibits neuroinflammation by up-regulating Aβ transporter activity to clear Aβ [227]. Aerobic exercise has been shown to alleviate the reduction of aging-induced cerebral blood flow (CBF) and cognitive performance in healthy individuals [228]. In addition, exercise also promotes the return of tight junction proteins in the BBB to their original levels, thereby restoring the permeability of the BBB. Long-term aerobic exercise from the midlife to old age prevents aging-related neurovascular decline, reduces the entry of inflammatory substances into the brain, and increases synaptic plasticity and overall behavioral capacity in aged mice [229]. Relevant studies have found that high-intensity continuous training (high-intensity interval training and high-intensity circuit training) has better immunomodulatory effects than moderate-intensity training [230]. Moreover, high-intensity circuit training is more effective in suppressing the proliferation of T cells and macrophages. In contrast, high-intensity interval training is better at inducing the anti-inflammatory phenotype polarization of immune cells [231]. Therefore, exercise suppresses neuroinflammation by altering the levels of peripheral inflammatory mediators and modulating microglia in AD models.

### Exercise promotes hippocampal neurogenesis through suppressing neuroinflammation

During adulthood, neural stem cells in the hippocampus of humans can continuously proliferate and differentiate to generate new neurons, termed adult hippocampal neurogenesis (AHN), which is closely related to the learning and memory capacity [232]. AHN exists only in the subventricular zone (SVZ) and subgranular zone (SGZ) in hippocampal tissues of the mammalian brain. AHN can promote brain plasticity in adulthood, and the newly generated neurons can re-establish the connections between damaged neurons [233]. Emerging evidence suggests that AHN is impaired prior to onset in classical mouse models of AD [234]. During the intraperitoneal injection of LPS in the brains of 3xTG mice, the number of immature neurons in the hippocampal tissues of 3xTG mice reveals the significant reduction when compared with wild-type mice, even leading to hippocampal-dependent memory loss [235]. The aggregation of Aβ leads to impaired function of neuronal stem cells in the adult hippocampus. Restoring stem cell function and reducing neuroinflammation are considered as the key therapeutic strategies for AD [236]. In addition, the immune system is an essential regulator of AHN. During the progression of AD, chronic inflammatory responses down-regulate AHN through anti-neurogenic effects. A study indicates that activated microglia, particularly the M1 phenotype, can promote inflammatory responses by reducing the survival of neural precursor cells and play an essential role in suppressing AHN [236]. It has been reported that intra-cerebroventricular injection of STZ in rats induces persistent neuroinflammatory responses in the hippocampal SVZ and SGZ, and inhibits the proliferation, differentiation, and maturation of neural precursor cells, thus leading to memory loss [237]. Extensive evidence confirms that exercise can promote the generation of new neurons in the lateral ventricle and the differentiation of immature neurons in young, middle-aged, and elderly subjects [238, 239]. Experiments show that 4 months of voluntary wheeling running promotes hippocampal neurogenesis in AD model mice [240]. Furthermore, exercise stimulates the secretion of selenoprotein for promoting the proliferation, differentiation and migration of hippocampal precursor cells [241]. However, drug-induced AHN does not affect cognitive function in 5xFAD mice, and only exercise-induced AHN can ameliorate cognitive impairment in mice [242]. According to previous reports, exercise can awaken dormant neural stem cells and clear senescent neural stem cells to enhance AHN and reverse Aβ-induced cognitive impairment, which may be related to BDNF [243–245]. Chronic inflammation impairs neural stem cell function, and the administration of NSAID inhibits LPS-induced systemic inflammatory responses in mice, elevates IL-6 level, and enhances neurogenesis [246]. The activation of inflammatory response-mediated glial cells and chemokines in AD can inhibit AHN in the brain and promote the pathological process of AD, especially at the later stage of AD. However, reducing chronic inflammation in AD-transgenic mice can increase the proliferation of hippocampal stem cells and delay the occurrence and progression of AD [236]. In addition, exercise can restore the damaged nerve regeneration mediated by neuroinflammation through PGC-1α/FNDC5/BNDF signal pathway, thereby inducing sympathetic activation and the generation of uric acid [247, 248]. The newly generated neurons can restore standard memory-storing neural circuits, increase the number of neuronal dendritic spines, and restore regular expression of some neuronal genes [249]. However, recent animal studies have found that the differentiation tendency of neural stem cells is related to the conditions of exercise [250]. For example, for a long-term runner, neural stem cells are more inclined to differentiate into astrocytes, and the self-proliferation capacity of microglia is also enhanced. Glial cells have a particular regeneration capacity and can be induced to reprogram or convert into neurons, thereby extensively replenishing damaged neurons [251]. Therefore, exercise suppresses inflammation and increases the number of neurons and glial cells in the hippocampus to repair the irreversible damage caused by inflammation, which may be essential for early prevention and improvement of AD.

## Exercise, neuroinflammation and neurodegenerative diseases

Numerous studies have shown that neuroinflammation is a common feature of neurodegenerative diseases [83]. A pathological hallmark of age-related degenerative diseases is the accumulation of excessive mis-folded proteins in neurons. These diseases cover tauopathy dominated by AD and synucleinopathies represented by Parkinson's disease (PD) and dementia with Lewy bodies [252]. These abnormal proteins appear to share the features such as the formation and insolubility of amyloid fibril structures that make them less susceptible to clearance by defense mechanisms in the body and induce the conversion of normal proteins to irregular forms in a prion-like manner [253–256]. The constantly emerging and accumulating erroneous proteins are likely the results of glial-neuron interactions in neuroinflammation. In addition, the studies on serial pathological section observations of postmortem brain tissue have found that the accumulation of these abnormal proteins is closely related to clinical symptoms and spreading in the brain in a specific way [257–259]. In various neurodegenerative disease models, exercise increases the number of anti-inflammatory phenotypes of microglia, thereby reducing the formation of faulty proteins, ultimately delaying disease progression and mitigating symptoms of neurodegenerative diseases, which also may be also related to the down-regulation of pattern recognition receptors in microglia and neuronal apoptosis [213, 260–263]. Likewise, the propagation of pathologically faulty proteins across various brain regions can cause the loss of differentiated mature neurons and impair the regeneration capacity of new neurons [264]. An experiment with autopsy specimens has demonstrated that increased susceptibility to AHN in different neurodegenerative diseases, suggesting that the functional decline of hippocampal neural stem cells may underlie cognitive impairment during pathological aging in humans [265]. In addition, abnormal glial function is associated with fragile AHN in aging and neurodegenerative diseases [265]. Neuroinflammation can effectively inhibit AHN, and exercise can accelerate the formation of new neurons, thereby resisting the damage caused by the inflammatory response and ultimately improving cognitive capacity [266]. Therefore, the role of exercise in immunomodulation and the repairing of damaged neurons appear to be informative in studying other neurodegenerative diseases. However, the causal relationship between exercise and neuroinflammation in neurodegenerative diseases has been less studied.

## Limitations and future directions

Possibly due to ethical restriction and the difficulties in obtaining human brain tissue samples, there are only a few human studies on the relationship between exercise and inflammation in AD, mainly based on animal experiments. Moreover, animal models used to explore the underlying mechanisms for neuroinflammation of AD vary widely. Even exercise has many negative results for AD and inflammation [267–269], because studies on exercise and inflammation in AD are still sparse, our current understanding of the effects of exercise on regulating inflammation and the role of inflammatory responses in AD is limited. Moreover, the exercise conditions are not the same in animal or human experiments. Therefore, in terms of the current research results, we raise some thought-provoking questions and views: which chronic neuroinflammation level can induce AD? Is Aβ as a bystander in AD neuroimmune responses? Whether the restoration of inflammation can reduce the prevalence of AD or improve the prognosis of patients with MCI and AD is unclear. What kind of exercise has the best anti-inflammatory effect for AD patients is not defined? Does exercise have immunomodulatory effects on non-polarized state, or non-M1/M2 phenotypes of microglia? Whether microglia and their intracellular NLRP3 are potential therapeutic targets for immunology-based drugs or biomarker development still needs to be clarified. The roles of astrocytes in neuroinflammation still need to be explored. Which "exerkines" are involved in the anti-inflammatory effects of exercise? Are the anti-inflammatory effects of exercise sustained throughout the lifespan? Does physical exercise affect plasma GFAP or TREM2 levels in subjects with MCI or AD? Is the anti-inflammatory effect of exercise on other neurodegenerative diseases? All above unsolved questions should be the future directions for the prevention and treatments of AD through exercise interventions, which are also highly needed for the resolutions based on animal or cell experiments or the identification of potential biomarkers for clinical trials or practice.

## Conclusion

Although exercise has been considered as an essential strategy for the prevention and treatment of AD, the specific mechanisms for ameliorating AD upon exercise interventions are still not fully uncovered, which is not conducive to further in-depth studies. Recent evidence highlights a more significant role of neuroinflammatory responses in AD pathogenesis, even prior to Aβ deposition, unlike previous amyloid cascade hypotheses. Therefore, this article explores whether exercise can prevent and treat AD by suppressing inflammatory response, summarizes the key features and possible mechanisms of inflammatory response in AD, and the relationship between the immune regulation of exercise and the role of promoting AHN and neuroinflammation.

## Data Availability

Not applicable.

## Abbreviations

AD: Alzheimer’s disease
AHN: Adult hippocampal neurogenesis
ALR2: AIM2-like receptor 2
APOEε4: Apolipoprotein E ε4
APP: Amyloid precursor protein
AQP4: Aquaporin 4
ASC: Apoptosis-associated speck-like protein containing a caspase recruitment domain (CARD)
Aβ: Amyloid-β peptide
BACE1: β-Site amyloid precursor protein cleaving enzyme 1
BBB: Blood–brain barrier
BDNF: Brain-derived neurotrophic factor
BiP: Immunoglobulin heavy-chain-binding protein
CBF: Cerebral blood flow
CNS: Central nervous system
CRP: C-reactive protein
CSF: Cerebrospinal fluid
DAM: Disease-associated microglia
DAMP: Danger-associated molecular pattern
EOAD: Early-onset family AD
ERS: Endoplasmic reticulum stress
GFAP: Glial fibrillary acidic protein
IFN-G: Interferon-G
IL-10: Interleukin 10
IL-17: Interleukin-17
IL-1RA: IL-1 Receptor antagonist
IL-1β: Interleukin-1 beta
IL-6: Interleukin-6
IRE1: Inositol-requiring enzyme 1
JNK: C-Jun N-terminal kinase
LOAD: Late-onset AD
LPS: Lipopolysaccharide
MAPK: Mitogen-activated protein kinase
MCI: Mild cognitive impairment
NBP: Dl-3-n-butylphthalide
NFT: Neurofibrillary tangle
NF-κB: Nuclear factor kappa-B
NLRP3: NOD-like receptor protein 3
NO: Nitric oxide
NR: Nicotinamide riboside
Nrf2: Nuclear factor-related factor 2
NSAID: Non-steroid anti-inflammatory drug
PAMP: Pathogen-associated molecular pattern
PERK: PKR-like ER kinase
PET: Positron emission tomography
PPR: Pattern recognition receptor
RNS: Reactive nitrogen species
ROS: Reactive oxygen species
SGZ: Subgranular zone
SOD: Superoxidase dismutase
SP: Senile plaque
SR-A: Scavenger receptor A
sTNFR1: Soluble tumor necrosis factor receptor-1
sTNFR2: Soluble tumor necrosis factor receptor-2
STZ: Streptozotocin
SVZ: Subventricular zone
TGF-β1: Transforming growth factor beta-1
TJ: Tight junction
TNF-α: Tumor necrosis factor alpha
TREM2: Triggering receptor expressed on myeloid cell 2
TRX: Thioredoxin
TXNIP: Thioredoxin-interacting protein
URP: Unfolded protein response
WHO: World Health Organization
